# Supplementary material for: Deletion of wfs1 Impairs Oligodendrocyte Precursor Cells Dorsal Distribution and Myelination Through the wfs1-hmgcs1 Axis in Zebrafish
Source: Biology (Basel). 2026 Aug 16;15(16):1402. doi: 10.3390/biology15161402 (PMC13510151; doi:10.3390/biology15161402)
Supplement: Supplementary file 1 [file biology-15-01402-s001.zip › File S1-Transcriptome sequencing data and differentially expressed genes (DEGs) in wfs1-- zebrafish at 5 dpf/2.KEGG/wfs1KOvsWT/ALL/src/dre04514.html]

dre04514


- dre:30194

- Up regulated genes

30194(1.89942872614741)

- dre:557935

- Down regulated genes

557935(-1.87319484154862)

- dre:541474

- Up regulated genes

541474(1.62928618286673)

- dre:541474

- Up regulated genes

541474(1.62928618286673)

- dre:541474

- Up regulated genes

541474(1.62928618286673)

- dre:541474

- Up regulated genes

541474(1.62928618286673)

- dre:541474

- Up regulated genes

541474(1.62928618286673)

- dre:30634

- Up regulated genes

30634\_1(4.53582791300907)

- dre:30634

- Up regulated genes

30634\_1(4.53582791300907)

- dre:30634

- Up regulated genes

30634\_1(4.53582791300907)

- dre:30634

- Up regulated genes

30634\_1(4.53582791300907)

- dre:797491

- Down regulated genes

797491\_1(-1.09418588609297)

- dre:797651

- Up regulated genes

797651\_1(1.84553757065454)

- Down regulated genes

797651(-1.27962292749959)

- dre:797651

- Up regulated genes

797651\_1(1.84553757065454)

- Down regulated genes

797651(-1.27962292749959)

- dre:557935

- Down regulated genes

557935(-1.87319484154862)

- dre:100005566

- Up regulated genes

100005566(1.73719164936456)

- dre:100005566

- Up regulated genes

100005566(1.73719164936456)

- dre:100005566

- Up regulated genes

100005566(1.73719164936456)

- dre:100005566

- Up regulated genes

100005566(1.73719164936456)

- dre:797651

- Up regulated genes

797651\_1(1.84553757065454)

- Down regulated genes

797651(-1.27962292749959)

- dre:562934

- Up regulated genes

562934\_1(4.15852381132488)

- dre:797651

- Up regulated genes

797651\_1(1.84553757065454)

- Down regulated genes

797651(-1.27962292749959)

- dre:100005566

- Up regulated genes

100005566(1.73719164936456)

- dre:557935

- Down regulated genes

557935(-1.87319484154862)

- dre:557935

- Down regulated genes

557935(-1.87319484154862)

- dre:30762

- Up regulated genes

30762(1.51549567324805)
- dre:368878

- Up regulated genes

368878(2.50701188401484)
- dre:30763

- Up regulated genes

30763(5.58822372755117)
- dre:571282

- Up regulated genes

571282(1.90251155561911)

- dre:100151365

- Up regulated genes

100151365\_1(4.45902602692177)

- dre:557935

- Down regulated genes

557935(-1.87319484154862)

- dre:100151365

- Up regulated genes

100151365\_1(4.45902602692177)

- dre:557935

- Down regulated genes

557935(-1.87319484154862)

- dre:562934

- Up regulated genes

562934\_1(4.15852381132488)

- dre:100005566

- Up regulated genes

100005566(1.73719164936456)

- dre:569084

- Down regulated genes

569084\_1(-2.40277205964249)
- dre:81585

- Down regulated genes

81585(-1.74514319517014)
- dre:60635

- Down regulated genes

60635\_1(-1.07799587140666)
- dre:110438642

- Down regulated genes

110438642(-3.83724028917273)
- dre:793143

- Up regulated genes

793143(6.32099639946357)

- dre:569084

- Down regulated genes

569084\_1(-2.40277205964249)
- dre:81585

- Down regulated genes

81585(-1.74514319517014)
- dre:60635

- Down regulated genes

60635\_1(-1.07799587140666)
- dre:110438642

- Down regulated genes

110438642(-3.83724028917273)
- dre:793143

- Up regulated genes

793143(6.32099639946357)

- dre:100005566

- Up regulated genes

100005566(1.73719164936456)

- dre:569084

- Down regulated genes

569084\_1(-2.40277205964249)
- dre:81585

- Down regulated genes

81585(-1.74514319517014)
- dre:60635

- Down regulated genes

60635\_1(-1.07799587140666)
- dre:110438642

- Down regulated genes

110438642(-3.83724028917273)
- dre:793143

- Up regulated genes

793143(6.32099639946357)

- dre:30762

- Up regulated genes

30762(1.51549567324805)
- dre:368878

- Up regulated genes

368878(2.50701188401484)
- dre:30763

- Up regulated genes

30763(5.58822372755117)
- dre:571282

- Up regulated genes

571282(1.90251155561911)

- dre:100005566

- Up regulated genes

100005566(1.73719164936456)

- dre:797651

- Up regulated genes

797651\_1(1.84553757065454)

- Down regulated genes

797651(-1.27962292749959)

- dre:557935

- Down regulated genes

557935(-1.87319484154862)

- dre:569084

- Down regulated genes

569084\_1(-2.40277205964249)
- dre:81585

- Down regulated genes

81585(-1.74514319517014)
- dre:60635

- Down regulated genes

60635\_1(-1.07799587140666)
- dre:110438642

- Down regulated genes

110438642(-3.83724028917273)
- dre:793143

- Up regulated genes

793143(6.32099639946357)

- dre:101886406

- Down regulated genes

101886406(-1.56097230660967)

- dre:100151365

- Up regulated genes

100151365\_1(4.45902602692177)

Close
